# Toddler Temperament Mediates the Effect of Prenatal Maternal Stress on Childhood Anxiety Symptomatology: The QF2011 Queensland Flood Study

**DOI:** 10.3390/ijerph16111998

**Published:** 2019-06-05

**Authors:** Mia A. McLean, Vanessa E. Cobham, Gabrielle Simcock, Sue Kildea, Suzanne King

**Affiliations:** 1 Mater Research Institute-University of Queensland, Brisbane, QLD 4072, Australia; mia.mclean@uq.edu.au (M.A.M.); v.cobham@psy.uq.edu.au (V.E.C.); gsimcock@usc.edu.au (G.S.); sue.kildea@mater.uq.edu.au (S.K.); 2 School of Psychology, The University of Queensland, Brisbane, QLD 4072, Australia; 3 Sunshine Coast Mind and Neuroscience Thompson Institute, University of Sunshine Coast, Sippy Downs, QLD 4556, Australia; 4 School of Nursing, Midwifery, and Social Work, The University of Queensland, Brisbane, QLD 4072, Australia; 5 Schizophrenia and Neurodevelopmental Disorders Research, Douglas Mental Health University Institute, Verdun, QC H4H 1R3, Canada; 6 Department of Psychiatry, McGill University, Montreal, QC H3A 1A1, Canada

**Keywords:** prenatal stress, natural disasters, internalizing behaviors, childhood anxiety, toddlerhood, temperament characteristics

## Abstract

It is not known whether alterations to temperamental characteristics associated with prenatal maternal stress (PNMS) exposure account for the development of childhood anxiety symptomatology (internalizing behaviors and anxiety symptoms). The QF2011 Queensland flood study examined whether (1) toddler temperamental characteristics explained the association between PNMS exposure and childhood anxiety symptomatology; and (2) whether effects were dependent upon child sex or the timing of gestational exposure to PNMS. We investigated the effects of various aspects of flood-related stress in pregnancy (objective hardship, cognitive appraisal, subjective distress) on maternal report of 16-month toddler temperament (attentional control, shy-inhibition, negative reactivity), 4-year maternal-reported childhood anxiety symptomatology (internalizing and anxiety symptoms; *N* = 104), and teacher reports of internalizing behaviors (*N* = 77). Severity of maternal objective hardship during pregnancy and shy-inhibited behaviors were uniquely associated with 4-year child anxiety symptoms. Mediation analyses found that higher levels of 16-month negative reactivity accounted, in part, for the relationship between increased maternal objective flood-related hardship and greater internalizing behaviors (maternal but not teacher report). Neither child sex nor gestational timing of exposure moderated the hypothesized mediations. Our findings highlight several pathways through which varying aspects of disaster-related PNMS may influence early childhood anxiety symptomatology.

## 1. Introduction

Internalizing behaviors (i.e., behaviors focused inward, including anxiety, depression, somatic complaints, and withdrawal) constitute the most common mental health problems during childhood. Anxiety disorders are the most common form of internalizing disorders experienced in preschool [1], with internalizing behavior rating scales routinely used as measures of child anxiety [2,3]. Approximately 20% of four- to seven-year-old children experience anxiety disorders [4], and similar prevalence rates for internalizing behaviors have been reported in school-age children [5,6]. In the current study, we aimed to further our understanding of the etiology of childhood anxiety symptoms and internalizing behaviors (collectively termed “anxiety symptomatology”) by examining outcomes in relation to various within-individual temperamental precursors in addition to a specific prenatal environmental factor: disaster-related prenatal maternal stress.

### 1.1. Temperament and Anxiety Symptomatology

Researchers have long recognized individual differences in temperamental characteristics as important vulnerability markers for the onset of anxiety symptomatology in early childhood and adolescence [7,8,9]. Internalizing behaviors and anxiety symptoms may have shared but also unique temperamental precursors. The presence of more shy-inhibited behaviors (fearful withdrawal from unfamiliar people, displays of shyness) and associated behavioral inhibition (withdrawal and fear in novel and/or unfamiliar situations) are most consistently related to more severe anxiety in later childhood [4,10,11,12], particularly social anxiety [13]. Associations between shy-inhibited temperament and later internalizing behaviors have also been established [7,14]. Empirical studies suggest that negative reactivity characteristics (anger, distress at limitations, moodiness, irritability) in toddlerhood are strongly associated with the later development of broader internalizing behaviors [9,15,16] and less so with later anxiety symptoms [12]. Finally, attentional focusing and shifting (attentional control), a regulatory behavior, may play a smaller direct role in both internalizing behaviors [17,18] and anxiety disorders in childhood [19,20].

An alternative way of considering these relationships involves the possibility that dysregulated temperamental characteristics may interact within an individual in a risk and resiliency fashion, leading to the development of anxiety symptomatology [21]. For example, better attentional control may help negate a highly reactive toddler’s automatic attention bias to threat by drawing their attention away from the threatening or worrying stimuli. This allows the toddler to engage in prosocial behaviors, which are predictive of better adjustment [17]. Studies suggest that in early childhood, greater negative emotionality (fear, irritability, distress) may only be associated with increased internalizing behaviors and anxiety symptoms in children with low levels of attentional control and associated self-regulatory behaviors (orientation duration and later, effortful control) [18,22].

### 1.2. The Role of Prenatal Maternal Stress

Prenatal maternal stress (PNMS) is proposed to affect infant temperament and later behavioral outcomes via the alteration of fetal neural systems involved in regulation and reactivity (e.g., the hypothalamic–pituitary–adrenal (HPA) axis, the amygdala), commonly known as fetal programming [23]. A number of studies have linked various aspects of maternal mood (depression, anxiety, pregnancy specific anxiety) as well as stress in reaction to life events during pregnancy to altered infant reactivity and regulatory behaviors [24]. Child and adolescent anxiety symptomatology have also been linked to PNMS exposure [25].

Findings from prospective longitudinal cohorts show the unique effects of disaster-related PNMS on both infant and toddler temperament, as well as on anxiety symptomatology [26,27,28,29]. Natural disaster PNMS studies allow for a thorough examination of the unique effects of various aspects of the stressful experience (including subjective post-traumatic stress-like symptoms, objective hardship due to the stressor, and cognitive appraisal of the impact of the event) on child development. Importantly, the natural experiment design reduces the likelihood that any associations identified are independent of mother–child genetic inheritance and other co-occurring risk factors, such as socioeconomic status [30,31]. Further, due to the sudden onset nature of disasters, the potentially differential effects of the timing of stressor exposure during gestation can be examined in relation to child outcomes.

Research with the current cohort, the QF2011 Queensland flood study, showed that at 6 months, lower levels of maternal objective hardship were associated with greater irritability in girls than boys. At higher levels of objective hardship, boys displayed greater irritability than girls. No effect of PNMS on early shy-inhibited behaviors was established [26]. At 4 years, QF2011 children whose mothers experienced greater flood-related objective hardship in early gestation experienced more severe anxiety symptoms and internalizing behaviors [28]. Infants exposed in-utero to Hurricane Sandy (‘Superstorm Sandy’) were more fearful and showed poorer attentional control at 6 months than un-exposed infants, yet fearfulness decreased from 6 to 24 months [29]. When investigated, PNMS effects on temperament or anxiety symptomatology were not dependent upon gestational timing of disaster exposure in any of these cohorts.

The effects of PNMS on temperament and anxiety symptomatology indicate that temperamental alterations may increase toddlers’ vulnerability to developing childhood psychopathology [24,32]. If this is the case, it offers a significant opportunity for targeted early intervention prior to the development of anxiety symptomatology. Two recent studies have examined whether temperamental negative emotionality mediated the association between types of prenatal maternal distress (significant life events and patterns of maternal mood) and child anxiety symptomatology [33,34]. In both studies, effects on child temperament and anxiety were independent, and no mediating effects of temperament between either aspect of maternal distress in pregnancy and anxiety nor internalizing were found. To date, no study has reported the role of toddler temperamental characteristics on the development of childhood anxiety symptoms in a disaster-related prenatally stressed cohort. In the current study, we seek to address this gap by examining fine-grained dimensions of toddler temperament when these characteristics are easily observable and more stable [35].

### 1.3. Child Sex

Another key objective is to examine the role of child sex in the development of early childhood anxiety symptomatology. Processes underlying fetal programming are often sex-specific [36], potentially due to placental functioning that differs between the sexes [37]. Maternal exposure to more stressful life events has been associated with increased fear responses [38] and internalizing symptoms in girls only [33]. Other research suggests that boys are more susceptible to PNMS exposure, displaying increased distress to limitations, poorer attentional control [39,40] and irritability [26] in infancy, as well as greater behavior problems in early childhood [41]. The investigation of the moderating factor of sex has potential implications for the etiology of anxiety in childhood and increased prevalence of symptoms [5,42,43,44] and associated neurobiological differences evident in females with anxiety and affective disorders [45]. In summary, we need further exploration of the potentially sex-specific development of anxiety symptomatology in PNMS cohorts.

### 1.4. The Current Study

In January 2011, 78% of the state of Queensland, Australia was declared a disaster zone due to the worst flooding in 35 years. The QF2011 Queensland flood study was established to prospectively track the immediate and long-term effects of aspects of flood-related PNMS on birth outcomes and development across childhood [46]. In the current study, we examined whether pathways of anxiety symptomatology development following in-utero exposure to varying levels of flood-related PNMS are explained by alterations to toddler temperamental characteristics.

**Figure 1:**
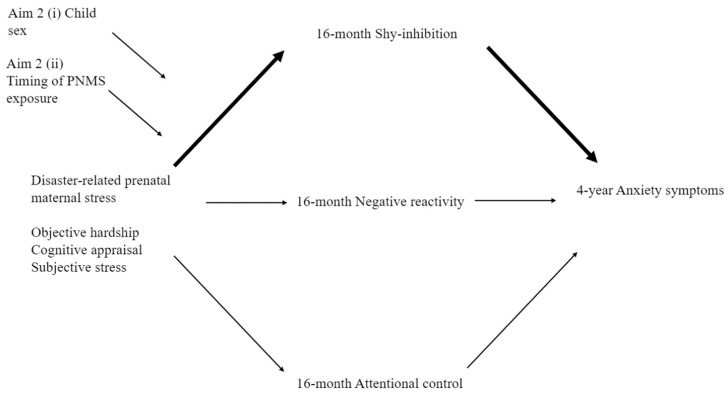
Conceptual diagram outlining the full, moderated mediation model of the indirect effects of disaster-related prenatal maternal stress (PNMS) on child anxiety symptoms as moderated by (a) child sex or (b) timing of exposure.

**Figure 2:**
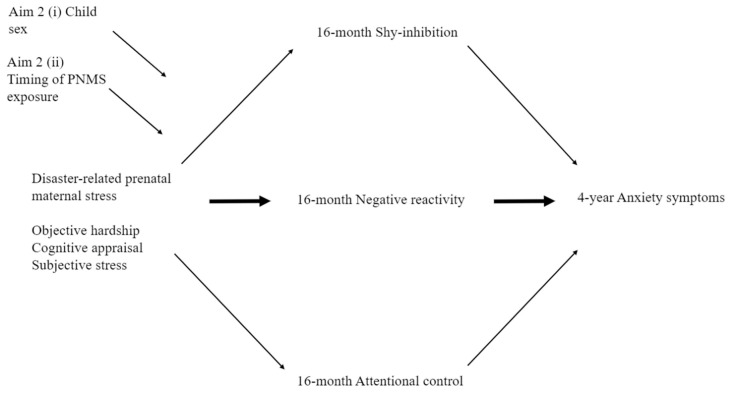
Conceptual diagram outlining the full moderated mediation model of the indirect effects of disaster-related prenatal maternal stress (PNMS) on child internalizing behaviors as moderated by (a) child sex or (b) timing of exposure.

The aims of the current study were:

**Aim 1.** To examine whether dimensions of toddler negative emotionality (reactivity and/or shy-inhibited) and regulatory behaviors (attentional control) mediate associations between disaster-related PNMS and early childhood anxiety symptomatology. Specifically, we hypothesized two indirect pathways:

(a) Anxiety symptoms. Greater exposure to maternal PNMS (objective, subjective, and/or cognitive appraisal) would result in greater toddler negative reactivity and/or shy-inhibited behaviors and/or poorer attentional control, which, in turn, would lead to higher levels of maternal-reported anxiety symptoms at four years. Given the extant literature, we hypothesized that shy-inhibited behaviors are most likely to mediate the pathway. See Figure 1.
(b) Internalizing behaviors. Greater exposure to maternal PNMS (objective, subjective, and/or cognitive appraisal) would result in greater toddler negative reactivity and/or shy-inhibited behaviors and/or poorer attentional control, which, in turn, would lead to higher levels of maternal- and/or teacher-reported internalizing symptoms. Given the extant literature, we hypothesized that negative reactivity behaviors are most likely to mediate the pathway. See Figure 2.

**Aim 2.** (i) To understand whether the effects shown in Figure 1 are dependent on the child’s sex and/or timing of exposure to PNMS. Based on previous findings [26,40], we hypothesized that (a) boys may display greater negative reactivity and, therefore, more internalizing behaviors, while (b) girls may display greater shy-inhibited behaviors prior to developing (b) anxiety symptoms. (ii) Due to the limited prior findings regarding the moderating role of timing of PNMS exposure on (a) anxiety symptoms and (b) internalizing behaviors, this aim was exploratory. See Figure 1 and Figure 2.

**Aim 3.** To examine whether temperamental reactivity and regulation characteristics interact to predict child anxiety symptomatology in the disaster-exposed PNMS cohort. Prior research suggests attentional control may moderate the effects of reactive temperamental styles [18]. Following initial mediation models, we also explored whether attentional control would buffer the effects of PNMS-influenced reactive temperamental characteristics (negative reactivity and/or shy-inhibited behaviors; Aims 1 and 2) on anxiety and/or internalizing behaviors.

## 2. Materials and Methods

### 2.1. Participants

From April 2011 (when ethical approval was received) to January 2012 (one year post-flood), we recruited 230 English-speaking women who were pregnant and at least 18 years of age, at the peak of the flood. All women provided written, informed consent for each part of the study. The study had ethical approval from Mater Research (1844M) and The University of Queensland (2013001236). Our protocol paper details information regarding participant recruitment, response rates, and recruitment sample characteristics [46]. There were two final samples used in analyses after we excluded infants born at birthweights under 2500 g and infants born earlier than 36 weeks’ gestation (*n* = 2 for the maternal reports, *n* = 1 for teacher-report sample). The maternal-report sample included a total of 104 mother–child dyads who had complete data at recruitment, maternal reports of temperament at 16 months (all dimensions), and 4-year anxiety symptoms and internalizing behaviors. The second sample (herein referred to as the “teacher-report sample”) consists of 77 mother–child dyads with maternal report of temperament at 16 months and 4-year teacher-reported internalizing behaviors.

### 2.2. Measures

#### 2.2.1. PNMS

Mothers reported on three aspects of PNMS at recruitment (reported between 3 and 12 months after the flood) and/or 12 months post-flood. For the maternal-report sample (Spence childhood anxiety scale for preschoolers (SPAS) and child behavior checklist (CBCL); *N* = 106 complete data prior to applying study criteria exclusions), a total of 37 mothers (34.9%) completed surveys at recruitment only and 47 mothers (44.3%) completed surveys at recruitment and 12 months post-flood. The remaining 22 mothers (20.8%) reported on flood-related PNMS on the 12-month post-flood survey only. For the teacher-report sample (*N* = 78 complete data prior to applying study criteria exclusions), 12 mothers (15.4%) reported at recruitment, 46 mothers (58%) reported at recruitment as well as 12 months post-flood, while 20 mothers (25.6%) reported at 12 months post-flood. For mothers who completed both surveys, scores across both surveys were integrated to provide an overall score on each aspect of PNMS.

*Objective hardship* related to the flood was assessed using the Queensland flood objective stress scale (QFOSS). The measure was adapted from scales designed by our group for previous disaster studies [47,48]. QFOSS assessed information regarding the severity of hardship experienced under four dimensions of flood-related exposures: threat, loss, scope, and change. The items in each scale and their scoring are presented in the Supplementary Material in the published protocol [46]. Scores on items within each dimension were summed, with possible scores between 0 (*no impact*) and 50 (*extreme impact*), with a maximum overall QFOSS score of 200. Because the distribution was positively skewed, a natural log transformation was conducted to normalize the data.

To assess the mother’s *cognitive appraisal* of the overall impact of the event, mothers responded to the question, “If you think about all of the consequences of the 2011 Queensland flood on you and your household, would you say the flood has been…?” Mothers’ rated their appraisal of the event on a five-point scale from “*Very negative*” (−2) to “*Very Positive*” (+2). The variable was dichotomized into “*Negative/Very negative*” (0) and “*There were no consequences/Positive/Very positive*” (1), due to the narrow range of responses on this scale and to isolate a negative cognitive appraisal by the mother.

Mothers completed three measures of *subjective stress* related to the flood. Each measure used a five-point rating scale: 0 (*not at all true*) to 4 (*extremely true*). Post-traumatic stress disorder (PTSD) symptoms (e.g., severity of intrusive thoughts, hyperarousal, and avoidance) in reaction to the flood were assessed using the 22-item impact of event scale—revised (IES-R) [49]. The peritraumatic distress inventory (PDI) is a 13-item scale asking participants to retrospectively report and rate emotional and physical reactions that they had experienced during and immediately following a traumatic experience [50]. The 10-item peritraumatic dissociative experiences questionnaire (PDEQ) [51] measures peritraumatic dissociative reactions to a specific trauma. These measures demonstrate good psychometric properties [52,53], and the PDI is predictive of a PTSD diagnosis [54].

To reduce the number of variables in the analyses conducted, the total scores from all three measures of subjective stress were used to compute a composite score for mothers’ subjective stress (COSMOSS) via principal component analysis (PCA). The PCA-derived algorithm was: COSMOSS = (0.358 × IESR) + (0.397 × PDI) + (0.387 × PDEQ). The resulting standardized factor explained 76.68% of the overall subjective stress variance.

#### 2.2.2. Toddler Temperament

At 16 months of age, mothers reported on their toddlers’ temperament via the short temperament scale for toddlers (STST) [55], normed for the Australian population. Prior work has demonstrated the sound psychometric properties of this measure when normed for Australian populations [55]. Parents rated the occurrence of common toddler behaviors on a six-point Likert scale (1, *almost never*; 6, *almost always*). Six dimensions of infant temperament are assessed across 30 items: approach–withdrawal, rhythmicity, cooperation–manageability, activity–reactivity, persistence, and distractibility. Normed factor scores are calculated from raw scores on each dimension. Scores of +1 SD above the standardized mean on each dimension represent difficult temperamental qualities, with scores –1 SD below the standardized mean classified as easy temperamental qualities.

In the current study, we used a participant’s overall factor score on the “approach–withdrawal” subscale as a measure of *shy-inhibited* temperament (Prior et al., 2000) and used the “persistence” subscale to measure *attention control* [12]. A measure of *negative reactivity* was created by averaging factor scores on the “cooperation–manageability” and the “reactivity” subscales. Prior empirical studies have examined these temperamental dimensions, using the subscales of the STST as we have done here [12,56,57].

#### 2.2.3. Child Anxiety Symptoms.

The 34-item Spence childhood anxiety scale for preschoolers (SPAS; [58]) provides an overall maternal-report measure of specific anxiety symptoms. The first 28 items ask parents to report the frequency at which an item is true for their child, from 1 (*not at all*) to 5 (*very often true*). The overall total score of anxiety symptoms was calculated and used for the analyses. Established cut-off scores classify children as within normal (*total score* ≤ 33) and elevated (*total score* ≥ 34) symptom ranges. The SPAS total anxiety score shows good construct validity with the CBCL-internalizing scale [58].

#### 2.2.4. Child Internalizing Behaviors

At four years, mothers and daycare/kindergarten teachers reported on the children’s internalizing behaviors using the child behavior checklist 1½–5 years (CBCL 1½–5) [59] and the caregiver–teacher report form (C-TRF) [59], respectively. We used data from the internalizing scales of the CBCL 1½–5 and the C-TRF. The CBCL 1½–5 and the C-TRF include identical items, scales, scoring, and cut-off scores. Mothers and teachers rate how true statements regarding the children’s behavior are, from 0 = *Not True*, to 2 = *Very True or Often True*. A CBCL-internalizing total *T* score (M = 50, SD = 10) was calculated as the sum of the responses to the 36 statements. Established standardized cut-off scores classify children as within normal (*T* ≤ 59), borderline (*T* ≥ 60 but ≤ 63), and clinical ranges (*T* ≥ 64). Both measures demonstrate strong psychometric properties and are routinely used as measures of child anxiety symptoms [2,3].

#### 2.2.5. Covariates

Based on the findings of our previous study examining four-year anxiety symptomatology in this cohort [28], we aimed to control for maternal factors that may influence child development and/or bias maternal reporting of child behavior [60]. Maternal six-month postpartum depression was assessed using the Edinburgh postnatal depression scale (EPDS) [61]. Higher scores (out of 40) indicated greater maternal postnatal depression symptoms. At 30 months, we measured maternal trait anxiety using the state-trait anxiety inventory (STAI-T) [62]. We included measures of concurrent mood (composite score of depression, anxiety, and stress subscales on the depression, anxiety, and stress scales short-form, DASS-21 [63]) at 16 months and 4 years. All four covariates (EPDS, STAI-T, and DASS-21 at 16 months and four years) were included as continuous covariates, with higher scores indicating greater symptoms of measured constructs. Child age at each assessment was also recorded.

#### 2.2.6. Demographics

Maternal socioeconomic status (SES), education level, and household income were measured at recruitment. Infant birth weight and gestational age were collected from medical records taken at birth.

### 2.3. Statistical Analysis

We conducted a series of parallel mediation analyses to explore Aim 1—whether the effects of objective hardship, cognitive appraisal, and subjective stress reactions on each measure of child anxiety symptomatology were mediated by the proposed temperamental characteristics (Figure 1). We assessed Aim 2, the moderating roles of (i) child sex and (ii) timing of exposure, via moderated mediation. Given the importance of understanding the role of PNMS on anxiety symptomatology in our study, Aim 3 was assessed using only the significant models established in Aims 1 and 2. Moderated mediation models were conducted by modifying the initial models, such that toddler attentional control might moderate the associations between negative reactivity and/or shy-inhibition on later anxiety symptomatology.

Analyses were conducted using the PROCESS Macro version 3.2 [64] allowing for mediation models to include control variables in their time-relevant paths (regression 1 or 2), given the longitudinal nature of the models. Bootstrap confidence intervals were generated for specific indirect effects, as well as for the indices of moderated mediation. In each model, objective hardship was either examined as a predictor or was controlled for when examining other aspects of maternal stress. Due to the relatively small sample, the relevant covariates established in our prior study [28] and the interaction terms forced into the equation that were nonsignificant at *p* < 0.10 in the final model were trimmed and the analyses were rerun. Simple slopes were used to probe significant interactions. Regression coefficients for parallel mediation models were standardized. For moderated mediation models, the coefficients were unstandardized.

Prior to testing the hypotheses as outlined, attrition analyses comparing complete and incomplete cases were conducted. Cross-period correlations were calculated, and scores for flood-related variables were finalized by integrating ratings provided at recruitment and/or 12 months post-flood using regression. Two outliers on the child anxiety symptom outcome variable (SPAS) were winsorized [65]. Missing data for covariates were imputed using expectation–maximization [66] techniques, given that data were found to be missing completely at random. Analyses were conducted using SPSS version 25 [67].

## 3. Results

### 3.1. Attrition Analyses

Results of independent t-tests showed that complete cases (those with data at recruitment, 16 months temperament, and 4 years prior to applying study criteria exclusions) of maternal-report anxiety symptomatology (*N* = 106, complete data prior to applying study criteria exclusions, i.e., premature infants) differed on one flood variable from incomplete cases (those who had any study relevant missing data; *N* = 124): timing of flood exposure. Complete cases experienced the floods later in gestation (M = 137.37 days; 19.62 weeks, SD = 78.43 days; 11.20 weeks) compared to incomplete cases (M = 98.55 days; 14.08 weeks, SD = 65.02 days; 9.29 weeks, *t*(204.40)) = 4.05, *p <* 0.001). Complete cases (mothers) were likely to have undertaken more years of schooling, on average (*N* = 105, M = 14.39 years, SD = 1.77 vs. *N* = 121, M = 13.59 years, SD = 2.08, *t*(225.96) = 3.16, *p* = 0.002) and were slightly older at the time of giving birth to their child than those with incomplete data (M = 31.87 years, SD = 4.50 vs. M = 30.37, SD = 5.82, *t*(225.87) = 2.20, *p* = 0.029).

### 3.2. Descriptive Statistics

Most children in the present study displayed temperamental characteristics and anxiety symptomatology within the normal range for their age (Table 1). Mother–child dyads were well resourced, with a mean socioeconomic index slightly higher than the national mean of 1000 (SD *=* 1000) across cohorts. Mothers were also well educated, with each cohort having an average of approximately 14 years of schooling.

### 3.3. Preliminary Analyses

Kolmogorov–Smirnov tests of normality suggested that objective hardship (log transformed) and negative reactivity were normally distributed for the maternal-report sample. For the teacher-report sample, only objective hardship (log transformed) was normally distributed. Table 2 and Table 3 display Pearson bivariate correlations between study variables for maternal-report and teacher-report samples, respectively. To better reflect parametric analyses used in mediation models (regression), we used Pearson’s correlations. Spearman rho correlations were also run due to the non-normality of data, with similar results.

All three PNMS maternal stress variables displayed moderate to high correlations for maternal and teacher reports of anxiety symptomatology; therefore, although all measures were somewhat related, they also assessed different aspects of the maternal stress response. Maternal and teacher report of child internalizing behaviors were uncorrelated (*r* = 0.06). Teacher report of internalizing behaviors showed a small positive correlation with maternal report of anxiety symptoms (*r* = 0.28), while maternal reports of internalizing and anxiety symptoms were highly correlated (*r* = 0.62). Descriptive statistics suggested that teachers may have scored children higher than mothers on internalizing behavior measures. However, repeated measure t-tests suggest that this was not the case (*t*(65) = 1.16, *p* = 0.249). No differences in flood variables, temperament or anxiety symptomatology across sample groups (mother only, teacher only, both mother and teacher report available) were found.

Objective PNMS was significantly correlated with SPAS anxiety (*r* = 0.22) and was marginally correlated with negative reactivity (*r* = 0.18) and CBCL-internalizing (*r* = 0.17). Subjective PNMS was marginally associated with CBCL-internalizing (*r* = 0.20). Shy-inhibited toddler behaviors showed a significantly positive correlation with both internalizing (*r* = 0.23) and anxiety symptoms (*r* = 0.22), while negative reactivity correlated positively with internalizing behaviors only (*r* = 0.36). As displayed in Table 3, scores on CTRF-internalizing were not correlated with any flood-related variable, study covariates or temperamental characteristic.

### 3.4. Main Analyses

#### 3.4.1. Aim 1

Indirect effects of PNMS variables on child anxiety symptomatology via toddler temperament.

**Figure 3:**
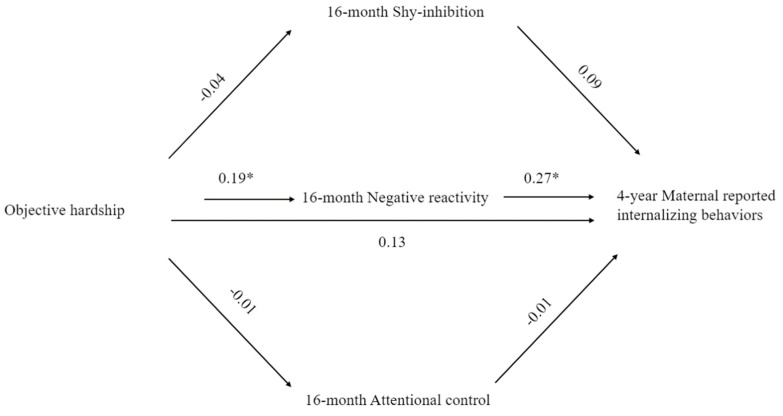
Mediation model displaying the indirect effect of objective hardship on maternal-reported child internalizing behaviors via negative reactivity at 16 months. All values are standardized beta coefficients. * *p <* 0.05.

(a) *Maternal Report of Anxiety Symptoms* (Table 4). The effect of objective stress on anxiety symptoms (SPAS) at age 4 years, after accounting for temperamental characteristics at 16 months, was positive and significant (β = 0.20, B = 2.38, *SE* = 1.06, *p* = 0.028, 95%CI (0.268, 4.494)). No significant indirect effects via temperamental attentional control, shy-inhibited behaviors or negative reactivity were found. Greater shy-inhibited behaviors predicted greater anxiety symptoms after controlling for exposure to prenatal objective hardship (β = 0.20, B = 2.04, *SE* = 0.98, *p* = 0.040, 95%CI (0.962, 3.98)). Exposure to the floods earlier in gestation tended to predict greater anxiety symptoms (β = −0.18, B = −0.02, *SE* = 0.01, *p* = 0.051, 95%CI (−0.420, 0.001)), independent of objective flood-related stress. The model accounted for 24.1% of variance in SPAS scores. Findings indicated that 4-year maternal mood and trait anxiety contribute substantially (10% variance) towards the presentation of child anxiety symptoms independent of temperament and flood-related effects. No significant direct or indirect pathways were uncovered when examining the role of prenatal subjective stress or cognitive appraisal as focal predictors.
(b) *Maternal Report of Internalizing Behaviors* (Table 4, Figure 3). A marginally significant mediation effect was found, whereby negative reactivity at 16 months mediated the effect of objective hardship on maternal-reported child internalizing behaviors at age 4 (mediation effect: β = 0.05, B = 0.71, *SE* = 0.03, 90%CI (0.004, 0.112)). Greater objective hardship was associated with greater negative reactivity at 16 months (β = 0.19, B = 0.13, *SE* = 0.06, *p* = 0.048), and greater negative reactivity was associated with greater internalizing behaviors at four years (β = 0.27, B = 5.47, *SE* = 1.76, *p* = 0.002) after accounting for relevant covariates (child age at the 16-month assessment, maternal mood at four years) and toddler attentional control and shy-inhibitory behaviors in the final model. The model accounted for 36% of variance in CBCL-internalizing scores, 20% of which was accounted for by maternal mood factors (postnatal maternal mood and 4-year concurrent mood). Again, no significant direct or indirect pathways were found when examining the role of subjective stress or cognitive appraisal as predictors.

*Teacher Report of Internalizing Behaviors.* No indirect or direct effects were established for PNMS effects when internalizing behaviors were reported by teachers (C-TRF).

#### 3.4.2. Aim 2

Moderation by (i) sex or (ii) timing of exposure of the indirect effect(s) of PNMS variables on child anxiety symptomatology via aspects of toddler temperament.

(a) *Maternal report of Anxiety Symptoms.* (i) For child sex, no significant moderated mediation pathways were uncovered when examining the role of maternal objective hardship, subjective stress or cognitive appraisal as predictors. (ii) Timing of exposure to the floods did not moderate mediation pathways of PNMS to anxiety symptoms via temperamental characteristics.
(b) *Maternal report of Internalizing Behaviors.* Neither (i) sex nor (ii) timing of exposure significantly moderated mediation pathways across any aspect of PNMS.

*Teacher report of Internalizing Behaviors.* No moderated mediation effects of (i) child sex or (ii) gestational timing of exposure were found for teacher reporting of internalizing behaviors (C-TRF) across any aspect of PNMS.

#### 3.4.3. Aim 3

Following the findings from Aim 1, we examined whether attentional control at 16 months moderated the pathway between negative reactivity at 16 months and CBCL maternal-report internalizing behaviors or anxiety symptoms within our model. The moderated mediation models were not significant.

## 4. Discussion

In a cohort of children whose mothers experienced a flood while pregnant, we sought to identify which individual temperamental characteristics may predispose a toddler to be more vulnerable to the development of childhood anxiety symptomatology. We found some support for the role of PNMS programming of early toddler temperamental negative reactivity characteristics, leading to the development of maternal- but not teacher-reported internalizing behaviors. By contrast, the role of PNMS on anxiety symptoms was independent of temperamental characteristics in toddlerhood. Given the small sample size used within the current study, results are interpreted with caution with replication of our findings recommended.

### 4.1. Pathways to Anxiety Symptoms and Internalizing Behaviors

Our findings suggest that pathways to childhood internalizing and anxiety symptoms may depend on temperamental characteristics in toddlerhood. This is consistent with prior literature. Shy-inhibited and associated constructs (e.g., fearfulness, behavioral inhibition) are most consistently linked to anxiety symptoms in childhood [13,56], while measures of negative reactivity are more consistently associated with the development of broader internalizing behaviors [9,15,16]. Further, anxiety symptoms only explain approximately 36% of the variance in internalizing behaviors within the current sample. From this, we can conclude that while anxiety and internalizing behaviors overlap, they are distinct constructs. It may be that temperamental characteristics are more strongly related to the development of conceptually related dimensions of psychopathology [68]. Our measure of negative reactivity incorporates aspects of moodiness and irritability akin to broader internalizing behaviors (depression, anxiety, withdrawal behaviors), whereas our measure of shy-inhibitory behaviors indicates a child’s distress towards novel situations (social situations) which may precede anxiety symptoms, particularly social anxiety symptoms [68]. We now discuss our findings in relation to each separate behavioral outcome.

### 4.2. Internalizing Behaviors

Greater flood-related objective hardship during pregnancy was uniquely related to more severe maternal-rated child internalizing behaviors via greater toddler negative reactivity. A stressful prenatal environment may prenatally program the toddler to expect a hostile postnatal environment, leading to increased toddler reactivity to threatening stimuli as an adaption to increase their survival [69]. Such alterations may, however, render children more vulnerable to experiencing internalizing problems via direct (e.g., expression of symptoms) and indirect effects (e.g., social interactions, parenting behaviors) [69,70].

Other studies have failed to establish a similar indirect path [33,34]. Discrepant findings to these studies also highlight the advantage of using methodologies that can disentangle the unique effect of different aspects of PNMS, independent of maternal–child heritability confounds on temperament and anxiety, and social selection bias. We found that it was the level of objective exposure, rather than subjective measures of maternal distress, that carried the effect in this and other studies [28,30]. The severity of objective flood hardship, regardless of maternal distress, may play a unique role in the temperamental vulnerabilities associated with internalizing behaviors. Importantly, our novel finding extends current developmental psychopathology models [68] by highlighting how one aspect of the prenatal maternal environment, but not others, may lead to negative reactivity, putting children at greater risk of developing internalizing behaviors.

As with previous work within the current cohort, we failed to establish effects of PNMS for internalizing behaviors as reported by teachers. Other PNMS studies report similar discrepancies [28,71,72]. In the current sample, maternal and teacher reports of internalizing behaviors were not correlated; however, scores on average did not vary across teacher and maternal report. Discrepancies between informants of child behavior is poorly understood [73,74]. Teachers observe child behavior in different contexts and interactions to that of mothers so may have a different impression of the child, varying reports across informants [75]. Unlike teachers, parents may see their child in various and diverse settings. Due to the logistical difficulty in obtaining teacher reports (as opposed to maternal reports), our sample size for this cohort was also smaller. Relevant to the current findings, future child psychopathology has been found to be better predicted by parents’ rather than teachers’ ratings [76].

### 4.3. Anxiety Symptoms

In line with prior research, we failed to establish a role for shy-inhibited behaviors or negative reactivity as mediators of PNMS effects on specific anxiety symptoms [34]. Rather, in line with a large body of work [13], shy-inhibited behaviors were associated with greater anxiety symptoms, independent of PNMS exposure. Greater objective hardship was uniquely related to the later development of more severe anxiety symptoms, independent of toddler temperament and maternal mood factors. Here, we replicate our previous findings within this cohort [28]. These findings are also consistent with previous findings within the QF2011 cohort, whereby PNMS had no effect on infant shy-inhibition at six months [26], suggesting PNMS effects on anxiety symptomatology may not first be identifiable via this behavioral trait. Still, it may be that effects of PNMS may be dormant and become more evident as the child develops, with continued tracking of our cohort necessary. In sum, our findings support the developmental psychopathology tenant of equifinality [77]: Displays of shy-inhibited temperament and PNMS exposure may independently and additively increase a child’s vulnerability to experiencing anxiety symptoms.

### 4.4. Attentional Control

Contrary to our hypotheses, disaster-related PNMS did not play a unique role in the development of toddler attentional control. Moreover, in contrast to prior research [18], toddler attentional control did not moderate the effects of reactive temperament traits on child anxiety symptomatology development. While a few studies have found that other types of PNMS play a role in the development of infant emotion regulation [39,78] and attentional control indices [40], others have also failed to establish similar associations [79]. It is possible that the effects of PNMS on attentional control abilities are more nuanced; they may be subtle and, therefore, identified later in development. Alternatively, PNMS effects may be dependent upon the type of maternal stress experienced, including maternal mood. Finally, postnatal factors not examined in the current study such as emotional support and parental sensitivity have been found to aid the development of aspects of emotion regulation and effortful control [80,81].

### 4.5. PNMS, Internalizing Behaviors and Anxiety Symptoms

In line with our prior work [28] the current study suggests that objective hardship, rather than subjective stress in pregnancy, may alter childhood anxiety and internalizing behaviors. The literature focuses on maternal–child mechanisms that may independently, and therefore, additively underlie the associations between the many various aspects of PNMS (anxiety, depression, stress) and child outcomes (e.g., maternal–child HPA axis, maternal inflammation, pregnancy nutrition, epigenetic changes) [82,83,84,85,86]. A more nuanced examination of the possible maternal–child mechanisms that underlie established associations between differential aspects of PNMS (objective hardship, subjective stress, maternal depression, anxiety) is clearly needed.

Our work suggests that, following disaster-related PNMS exposure, the pathways to internalizing behaviors and anxiety symptoms vary. Research suggests that prenatal maternal mood can affect fetal brain development; specifically the limbic structures (amygdala, hippocampus) [87] and the frontal lobe [88], which are areas involved in arousal, emotion regulation, and anxiety and internalizing disorders [89,90,91]. It is possible that objective PNMS affects fetal neurodevelopment in areas associated with temperamental negative reactivity and, independently, anxiety symptoms. Investigations of specific brain–behavior associations suggest there may be differences in amygdala connectivity patterns associated with fear and sadness during infancy and toddlerhood [92], and in youths experiencing irritability and anxiety [93]. The emerging research field of precision psychiatry may help us to understand the neural mechanisms underlying distinct and yet correlated behavior dimensions (e.g., negative reactivity versus anxious behaviors) associated with PNMS exposure.

Psychosocial factors could also account for the effects of PNMS on toddler negative reactivity, independent of anxiety symptoms. In the current study, it is evident that maternal postnatal mood and trait anxiety played a substantial role in the development of childhood anxiety symptomatology. While it is possible that PNMS effects could be attributed to the enduring effects of poor postnatal maternal mood [94], we found that the level of objective hardship experienced by mothers was unrelated to maternal mood throughout development. We initially included maternal mood in our proposed models primarily to account for maternal reporter bias of child behavior [60]. Mechanisms of maternal mood transmission are also possibly environmental via anxiety-maintaining parenting behaviors; [95,96] or genetic in nature [97,98]. Overall, our findings highlight the importance for observational PNMS research designs to account for maternal mood across development to disentangle the role of prenatal environmental factors from postnatal or enduring maternal factors.

### 4.6. Sex and Timing Effects

As with our previous examinations of this cohort, we failed to establish a moderating role of timing of PNMS on temperament [26] or anxiety [28]. Moreover, while sexually dimorphic responses to in-utero stress have been reported [32], we failed to establish sex differences. Sex differences in anxiety symptomatology due to PNMS may become more apparent in adolescence [99]. In most clinical populations, no sex differences in prevalence in the preschool period are evident [1]. Given the mixed findings across the literature, larger scale studies are needed to thoroughly examine both sex and timing of gestation as moderators.

Replicating our previous findings [28], we established a direct effect of the timing of exposure to the floods on anxiety symptoms, but not on internalizing behaviors. Children exposed to the flood earlier in pregnancy exhibited increased anxiety symptoms, independent of the level of objective hardship or subjective distress reported by their mothers. Aspects of PNMS unaccounted for by our measures may have affected biological processes in ascendance during early gestation (e.g., differentiation of the amygdala) [100,101]. Alternatively, studies suggest that children born in spring, as were those who experienced the southern-hemisphere January floods earlier in gestation, tend to display greater anxiety symptoms [102]. Our finding may, therefore, be the result of normal season of birth trends.

### 4.7. Limitations

A common problem of longitudinal studies, the small sample size in the current study due to attrition across time, is one clear limitation. The QF2011 cohort represents a relatively healthy population (higher SES), with mothers and children displaying largely nonclinical levels of stress and anxiety. Much of the sample displayed temperamental characteristics and anxiety symptomatology within the normal range. We were therefore unable to analyze the relative risk of clinically significant symptom development following PNMS exposure. Post-hoc power analyses suggested that our power to detect the marginally significant mediation effect of QFOSS on child internalizing behaviors via toddler temperament negativity was low (58.5%). The lack of variability in child behavioral scores, in addition to the small sample size used for mediation and moderated mediation analyses, may have reduced our power to detect other real changes due to PNMS. Still, we detected dose–response effects, suggesting a robust effect of PNMS within a natural experiment design on the development of internalizing behaviors. Moreover, the current results suggest that even with a larger sample size, highly nonsignificant PNMS effects are unlikely to be detected. While paternal mood during pregnancy and throughout child development [103,104] are thought to play a role in the development of childhood anxiety, given the difficulty of obtaining this data, we did not solicit participation from fathers. Our findings support the programming role of objective hardship due to an independent quasi-randomly distributed stressor during pregnancy, suggesting that data regarding paternal stress in pregnancy at least would likely not impact the genetic sensitivity of our findings.

### 4.8. Strengths

There are several strengths to the QF2011 study. Experiencing a natural disaster during pregnancy stresses the mother, her physiology, and the fetus to an extent that most PNMS studies lack. Although most PNMS studies include a wide variety of possible stressful life events that occur to women during pregnancy, QF2011 subjects all experienced the same stressor but to different degrees, providing a natural experiment with a dose–response component. The sudden-onset nature of the flood offered the ideal assessment of the timing of stress in pregnancy that is nearly impossible to do with other prenatal life events that have gradual onsets, such as the breakdown of a relationship, or even the death of a relative. While setting up a pregnancy study within weeks of a major disaster has many challenges, the difficulties outweigh the advantages of having data from a natural experiment. Importantly, when assessing child outcomes, we used both maternal and teacher reports of child anxiety symptomatology. Maternal reports have been correlated with observational measures in the literature [105], and maternal reporter bias was accounted for by controlling for concurrent maternal mood [60].

### 4.9. Future Research

There are several avenues for future research based on our current findings. We will continue to track temperament and anxiety symptom development in the QF2011 cohort to examine the long-term trajectory of these problems across middle childhood into adolescence, Moreover, the current study focused on examining behavioral markers of PNMS programming. PNMS effects may additionally or alternatively be evident via alterations to physiological anxiety-specific vulnerability factors in toddlerhood (e.g., cortisol reactivity) [106]. Indeed, the degree of overlap between temperamental traits, such as negative emotionality and physiological reactivity, is unclear [106], with each found to be uniquely and therefore additively associated with later anxiety symptomatology [107,108,109,110,111]. Future investigations of the role of self-regulatory abilities in the development of anxiety following PNMS should look to examine a broader range of indices, including cognitive, emotional, and behavioral constructs not included in the present study [78,112]. Finally, we encourage replication of our findings within larger prospective cohort studies, given the small sample size we used to undertake modelling of child developmental outcomes. Cumulatively, such work will contribute to a better understanding of who exactly we need to help in the wake of a natural disaster, when, and how it is best to do so.

## 5. Conclusions

In this study, we set out to examine the developmental pathways from PNMS to anxiety symptomatology via various toddler temperament characteristics. Our study has identified differential models for anxiety and internalizing behaviors following exposure to varying types of PNMS, thanks to our natural disaster methodology. Our empirical findings within our small cohort provide a more complete understanding of the complexity of possible developmental pathways to anxiety symptomatology when considering the prenatal environment. Together, our results support the developmental psychopathology tenants of equifinality and multifinality [76]: Multiple pathways lead to the development of anxiety symptoms and internalizing behaviors, while objective hardship may act via differential biopsychosocial mechanisms in the development of both outcomes. Our research joins an ever-growing body of work in developmental psychopathology suggesting that developmental processes involved in early childhood anxiety symptomatology begin prior to birth.

## Figures and Tables

**Table 1:** Cohort descriptive statistics for study demographics, predictor, and outcome variables.

Variables		Maternal-Report Sample *N* = 104	Teacher-Report Sample *N* = 77
Maternal Demographics			
Maternal age at childbirth	M (SD)	31.9 (4.5)	32.2 (4.9)
Socioeconomic Index ^a^	M (SD)	1054.1 (54)	1065.1 (49.1)
Schooling level (years)	N	103	77
	M (SD)	14.4 (1.8)	14.4 (2.0)
Maternal Mood Across Development			
6-month Maternal Depression ^b^	N	84	62
	M (SD)	6.3 (4.4)	5.8(4.0)
16-month Maternal Mood ^c^	N	104	77
	M (SD)	19.5 (15.8)	19.5 (15.7)
30-month Maternal Trait Anxiety ^d^	N	94	74
	M (SD)	39.7 (9.3)	38.81 (9.1)
4-year Maternal Mood ^c^	N	103	65
	M (SD)	20.1 (19.8)	18.9 (18.9)
Child Descriptives			
Gestational age at birth (wks)	M (SD)	39.5 (1.2)	39.4 (1.2)
Birthweight (grams)	M (SD)	3583.3 (466.5)	3576.4 (464.6)
Child Sex (boys)	N (%)	57 (54.8)	44 (57.1)
Child age at 16 months	M (SD)	16.5 (1.5)	16.3 (1.5)
Child age at 4 years	M (SD)	48.8 (1.3)	48.8 (1.6)
Flood-related Variables			
Objective hardship	M (SD)	20.9 (17.7)	23.4 (18.7)
Post-traumatic stress ^e^	M (SD)	5.7 (9.3)	6.2 (11)
Peritraumatic distress ^f^	M (SD)	12.3 (7.8)	12.1 (8.4)
Peritraumatic dissociation ^g^	M (SD)	6.01 (7.01)	6.1 (7.3)
Composite subjective stress ^h^	M (SD)	0.00 (.9)	0.01 (.1)
Cognitive Appraisal: Neg	N (%)	35 (33.7)	27 (35.1)
Cognitive Appraisal: Neut/Pos	N (%)	69 (66.3)	50 (65)
Timing of exposure (days)	M (SD)	137.8 (79.1)	147.8 (75.1)
16-month Toddler Temperament			
STST ^i^ Negative Reactivity Factor 16mo	M (SD)	3.54 (.53)	3.57 (0.54)
STST Approach Factor 16mo	M (SD)	3.07 (.91)	3.06 (1.03)
STST Persistence Factor 16mo	M (SD)	2.84 (.78)	2.90 (0.86)
4-year Anxiety Symptomatology			
CBCL ^j^-Internalizing T score	N	104	66
	M (SD)	44.2 (10.6)	43.9 (11.1)
CBCL-Internalizing Normal Range	N (%)	10 (90.4)	89.4 (59)
C-TRF ^k^-Internalizing T score	N	66	77
	M (SD)	46.1 (11)	46.5 (10.7)
C-TRF-Internalizing Normal Range	N (%)	60 (93.4)	69 (90)
SPAS ^l^ Anxiety Score	N	104	66
	M (SD)	12.2 (10.1)	11.9 (8.6)
SPAS Normal Range	N (%)	98 (95.2)	97 (64)

*Note.* Untransformed scores are used for the measures of maternal stress. ^a^ SEIFA, ^b^ Maternal depression: 6 month EPDS, ^c^ DASS (depression, anxiety, stress) composite, ^d^ STAI-Trait anxiety scale at 30 months, ^e^ = IESR, ^f^ = PDI, ^g^ = PDEQ, ^h^ = COSMOSS (IES-R, PDI, PDEQ), ^i^ = Short temperament scale for toddlers, ^j^ = CBCL = Child behavior checklist, ^k^ = score C-TRF = Caregiver–teacher report form, ^l^ = SPAS = Spence preschool anxiety scale.

**Table 2:** Pearson’s correlations between study variables for the maternal-report sample (*N* = 104).

Variables	1	2	3	4	5	6	7	8	9	10	11	12	13	14	15	16	17	18	19	20
1. SPAS ^a^ Total Score	-	-	-	-	-	-	-	-	-	-	-	-	-	-	-	-	-	-	-	-
2. CBCL ^b^ Internalizing T-score	0.62 **	-	-	-	-	-	-	-	-	-	-	-	-	-	-	-	-	-	-	-
3. CTRF ^c^ Internalizing T-score	0.28 *	0.06	-	-	-	-	-	-	-	-	-	-	-	-	-	-	-	-	-	-
4. STST ^d^ Negative Reactivity 16mo	0.17	0.36 **	0.10	-	-	-	-	-	-	-	-	-	-	-	-	-	-	-	-	-
5. STST ^d^ Shy-inhibition 16mo	0.22 *	0.23 *	0.02	0.30 **	-	-	-	-	-	-	-	-	-	-	-	-	-	-	-	-
6. STST ^d^ Persistence 16mo	0.02	0.11	−0.00	0.21 *	0.28 **	-	-	-	-	-	-	-	-	-	-	-	-	-	-	-
7. Objective hardship	0.22 *	0.17 ^	−0.05	0.18 ^	−0.04	−0.01	-	-	-	-	-	-	-	-	-	-	-	-	-	-
8. Post-traumatic stress ^e^	0.14	0.15	−0.09	0.23 *	−0.04	0.07	0.56 **	-	-	-	-	-	-	-	-	-	-	-	-	-
9. Peritraumatic distress ^f^	0.09	0.19 ^	−0.10	0.14	−0.02	−0.09	0.46 **	0.60 **	-	-	-	-	-	-	-	-	-	-	-	-
10. Peritraumatic dissociation ^g^	0.23 *	0.21 *	−0.04	0.11	0.08	−0.10	0.46 **	0.48 **	0.72 **	-	-	-	-	-	-	-	-	-	-	-
11. Composite subjective stress ^h^	0.16	0.20 *	−0.06	0.15	−0.00	−0.04	0.49 **	0.73 **	0.80 **	0.82 **	-	-	-	-	-	-	-	-	-	-
12. Cognitive appraisal ^i^	−0.02	−0.05	0.13	−0.04	0.12	0.16	−0.57 **	−0.45 **	−0.32 **	−0.25 *	−0.31 **	-	-	-	-	-	-	-	-	-
13. Timing of Exposure (days)	−0.15	−0.03	−0.02	0.13	0.12	0.14	0.01	0.12	0.06	0.08	0.10	0.06	-	-	-	-	-	-	-	-
14. Child sex	−0.14	−0.15	0.04	−0.09	0.04	0.10	−0.06	−0.07	−0.15 ^	−0.04	−0.13	0.07	−0.01	-	-	-	-	-	-	-
15. 6-month Maternal Depression ^j^	0.30 **	0.43 **	−0.07	0.12	0.17	−0.00	0.06	0.22 *	0.28 **	0.26 *	0.31 **	−0.16	−0.02	−0.08	-	-	-	-	-	-
16. 16-month Maternal Mood ^k^	0.20 *	0.36 **	0.13	0.03	−0.10	0.07	0.10	0.14	0.19 ^	0.25 **	0.23 *	−0.05	0.09	0.00	0.50 **	-	-	-	-	-
17. 30-month Trait Anxiety ^l^	0.32 **	0.36 **	0.12	0.21 *	0.14	0.18 *	0.10	0.19	0.25 *	0.27 *	0.27 *	−0.14	0.02	0.06	0.54 **	0.41 **	-	-	-	-
18. 4-year Maternal Mood ^k^	0.29 **	0.45 **	−0.10	0.05	0.12	0.11	−0.03	0.06	0.14	0.15 *	0.10	0.11	0.00	−0.01	0.62 **	0.64 **	0.45 **	-	-	-
19. 16-month age at assessment	−0.06	0.02	0.04	−0.18 ^	0.08	−0.20 *	0.10	−0.09	0.06	0.02	0.03	−0.16	−0.07	0.00	−0.21 ^	−0.06	−0.20 ^	−0.11	-	-
20. 4-year age at assessment	−0.01	−0.01	0.16	−0.14	−0.08	−0.05	−0.08	−0.01	0.14	0.13	0.13	0.06	0.20 *	−0.01	0.16	0.33 **	0.16	0.28 **	0.18 ^	-

*Note. N* = 104 for all correlations, other than C-TRF internalizing behavior correlations where *N* = 66. Transformed scores are used for the measures of maternal stress. Raw scores used for imputed covariates. ^a^ SPAS = Spence preschool anxiety scale, ^b^ CBCL = Child behavior checklist, ^c^ C-TRF = Caregiver–teacher report form, ^d^ = Short temperament scale for toddlers, ^e^ = IESR, ^f^ = PDI, ^g^ = PDEQ, ^h^ = COSMOSS (IES-R, PDI, PDEQ), ^i^ = Coding for cognitive appraisal: 0 = negative/very negative; 1 = neutral/ positive/very positive, ^j^ = Depression: 6-month EPDS, ^k^ = DASS (depression, anxiety, stress) composite score at 4 years, ^l^ = STAI-Trait anxiety scale at 30 months. ** *p* < 0.001; * *p* < 0.05; ^ *p* = 0.051–0.99.

**Table 3:** Pearson’s correlations between study variables for the teacher-report sample (*N* = 77).

Variables	1	2	3	4	5	6	7	8	9	10	11	12	13	14	15	16	17	18	19	20
1. SPAS ^a^ Total Score	-	-	-	-	-	-	-	-	-	-	-	-	-	-	-	-	-	-	-	-
2. CBCL ^b^ Internalizing T-score	0.58 **	-	-	-	-	-	-	-	-	-	-	-	-	-	-	-	-	-	-	-
3. CTRF ^c^ Internalizing T-score	0.28 *	0.06	-	-	-	-	-	-	-	-	-	-	-	-	-	-	-	-	-	-
4. STST ^d^ Negative Reactivity 16mo	0.17	0.39 **	0.11	-	-	-	-	-	-	-	-	-	-	-	-	-	-	-	-	-
5. STST ^d^ Shy-inhibition 16mo	0.17	0.36 **	−0.16	0.36 **	-	-	-	-	-	-	-	-	-	-	-	-	-	-	-	-
6. STST ^d^ Persistence 16mo	0.39 **	0.16	0.01	0.30 **	0.33 **	-	-	-	-	-	-	-	-	-	-	-	-	-	-	-
7. Objective hardship	0.12 ^	0.15	−0.04	0.11	−0.04	−0.11	-	-	-	-	-	-	-	-	-	-	-	-	-	-
8. Post-traumatic stress ^e^	0.24	−0.02	−0.08	0.15	−0.01	−0.01	0.57 **	-	-	-	-	-	-	-	-	-	-	-	-	-
9. Peritraumatic distress ^f^	−0.01	0.11	−0.15	−0.07	−0.07	−0.23 *	0.42 **	0.61 **	-	-	-	-	-	-	-	-	-	-	-	-
10. Peritraumatic dissociation ^g^	0.04	0.08	0.00	−0.04	0.08	−0.23 *	0.46 **	0.46 **	0.65 **	-	-	-	-	-	-	-	-	-	-	-
11. Composite subjective stress ^h^	0.14	0.01	−0.05	0.02	0.03	−0.22	0.48 **	0.75 **	0.79 **	0.80 **	-	-	-	-	-	-	-	-	-	-
12. Cognitive appraisal ^i^	0.08	0.04	0.14	0.03	0.08	0.16	−0.58 **	−0.48 **	−0.32 **	−0.26 *	−0.41 **	-	-	-	-	-	-	-	-	-
13. Timing of Exposure (days)	−0.21 ^	−0.07	−0.09	0.01	0.10	0.14	−0.01	0.17	0.12	0.04	0.08	0.06	-	-	-	-	-	-	-	-
14. Child sex	−0.09	−0.17	−0.02	−0.14	0.13	0.08	−0.01	−0.04	−0.07	0.03	−0.03	−0.02	0.00	-	-	-	-	-	-	-
15. 6-month Maternal Depression ^j^	0.19	0.39 **	−0.08	0.28 *	0.18	0.17	0.14	0.27 *	0.33 **	0.15	0.29 *	0.03	0.14	−0.16	-	-	-	-	-	-
16. 16-month Maternal Mood ^k^	0.08	0.29 *	0.11	0.15	−0.05	0.06	0.09	0.20 ^	0.16	0.17	0.19	0.02	0.11	−0.01	0.58 **	-	-	-	-	-
17. 30-month Trait Anxiety ^l^	0.34 **	0.32 *	0.08	0.30 **	0.14	0.23 ^	0.11	0.17	0.18	0.12	0.17	−0.05	0.10	−0.01	0.47 **	0.39 **	-	-	-	-
18. 4-year Maternal Mood ^k^	0.36 **	0.46 **	−0.16	0.11	0.21	0.09	0.04	0.06	0.17	0.19	0.09	0.26 *	0.03	0.01	0.51 **	0.57 **	0.47 **	-	-	-
19. 16-month age at assessment	0.03	−0.03	0.05	−0.17	0.14	0.06	0.03	−0.21 ^	−0.02	0.07	−0.02	−0.11	0.02	0.04	−0.21	−0.18	−0.23 ^	−0.17	-	-
20. 4-year age at assessment	−0.09	−0.11	0.14	−0.17	−0.10	−0.05	−0.06	−0.01	0.22^	0.19	0.18	0.14	0.31	0.02	0.09	0.25 *	0.13	0.14	0.13	-

*Note. N* = 77 for all correlations, other than SPAS anxiety and CBCL internalizing behavior correlations where *N* = 66. Transformed scores are used for the measures of maternal stress. Raw scores used for imputed covariates. ^a^ SPAS = Spence preschool anxiety scale, ^b^ CBCL = Child behavior checklist, ^c^ C-TRF = Caregiver–teacher report form, ^d^ = Short temperament scale for toddlers, ^e^ = IESR, ^f^ = PDI, ^g^ = PDEQ, ^h^ = COSMOSS (IES-R, PDI, PDEQ), ^i^ = Coding for cognitive appraisal: 0 = negative/very negative; 1 = neutral/ positive/very positive, ^j^ = Depression: 6-month EPDS, ^k^ = DASS (depression, anxiety, stress) composite score at 4 years, ^l^ = STAI-Trait anxiety scale at 30 months. ** *p* < 0.001; * *p* < 0.05; ^ *p* = 0.051–0.99.

**Table 4:** Trimmed final models: Standardized indirect effects of prenatal exposure to a natural disaster on maternal report of child anxiety symptomatology.

Direct and Indirect Effects	Value	90%CI (LLCI, ULCI)
*Spence Preschool Anxiety Scale-Anxiety* ^a^		
Objective Hardship -> Shy-inhibition -> SPAS-Anxiety	−0.009	(−0.051, 0.027)
Objective Hardship -> Negative Reactivity -> SPAS-Anxiety	0.012	(−0.023, 0.058)
Objective Hardship -> Attentional Control -> SPAS-Anxiety	0.008	(−0.018, 0.021)
*Child Behavior Checklist-Internalizing* ^b^		
Objective Hardship -> Shy-inhibition -> CBCL-Internalizing	−0.004	(−0.028, 0.015)
Objective Hardship -> Negative Reactivity -> CBCL-Internalizing	**0.053**	**(0.004, 0.112)**
Objective Hardship -> Attentional Control -> CBCL-Internalizing	0.001	(−0.012, 0.015)

^a^ Model controls for child age at 16-month assessment for negative reactivity (path a), maternal trait anxiety (path b), and concurrent maternal mood (path b). ^b^ Model controls for child age at 16-month assessment for negative reactivity (path a), 6-month maternal mood (path b), and 4-year maternal mood (path b). Note: Bolded values indicate marginally significant paths.
